# Clusters of lifestyle behavioral risk factors and their associations with depressive symptoms and stress: evidence from students at a university in Finland

**DOI:** 10.1186/s12889-024-18421-0

**Published:** 2024-04-22

**Authors:** Walid El Ansari, Rene Sebena, Kareem El-Ansari, Sakari Suominen

**Affiliations:** 1 Department of Surgery, Hamad General Hospital, Hamad Medical Corporation, 3050 Doha, Qatar; 2 College of Medicine, Qatar University, 3050 Doha, Qatar; 3 Department of Population Health, Weill Cornell Medicine-Qatar, Doha, Qatar; 4 Department of Psychology, Faculty of Arts, PJ Safarik University, Kosice, Slovak Republic; 5 Faculty of Medicine, St. George’s University, Saint George’s, Grenada; 6 School of Health Sciences, University of Skövde, 541 28 Skövde, Sweden; 7 Department of Public Health, University of Turku, Turku, Finland; 8 Research Services, The wellbeing services county of Southwest Finland, Turku, Finland

**Keywords:** Behavioral risk factors, Depressive symptoms, Stress, Mental health, University students, Cluster analysis

## Abstract

**Background:**

No previous research of university students in Finland assessed lifestyle behavioral risk factors (BRFs), grouped students into clusters, appraised the relationships of the clusters with their mental well-being, whilst controlling for confounders. The current study undertook this task.

**Methods:**

Students at the University of Turku (*n* = 1177, aged 22.96 ± 5.2 years) completed an online questionnaire that tapped information on sociodemographic variables (age, sex, income sufficiency, accommodation during the semester), four BRFs [problematic alcohol consumption, smoking, food consumption habits, moderate-to-vigorous physical activity (MVPA)], as well as depressive symptoms and stress. Two-step cluster analysis of the BRFs using log-likelihood distance measure categorized students into well-defined clusters. Two regression models appraised the associations between cluster membership and depressive symptoms and stress, controlling for sex, income sufficiency and accommodation during the semester.

**Results:**

Slightly more than half the study participants (56.8%) had always/mostly sufficient income and 33% lived with parents/partner. Cluster analysis of BRFs identified three distinct student clusters, namely Cluster 1 (Healthy Group), Cluster 2 (Smokers), and Cluster 3 (Nonsmokers but Problematic Drinkers). Age, sex and MVPA were not different across the clusters, but Clusters 1 and 3 comprised significantly more respondents with always/mostly sufficient income and lived with their parents/partner during the semester. All members in Clusters 1 and 3 were non-smokers, while all Cluster 2 members comprised occasional/daily smokers. Problematic drinking was significantly different between clusters (Cluster 1 = 0%, Cluster 2 = 54%, Cluster 3 = 100%). Cluster 3 exhibited significantly healthier nutrition habits than both other clusters. Regression analysis showed: (1) males and those with sufficient income were significantly less likely to report depressive symptoms or stress; (2) those living with parents/partner were significantly less likely to experience depressive symptoms; (3) compared to Cluster 1, students in the two other clusters were significantly more likely to report higher depressive symptoms; and (4) only students in Cluster 2 were more likely to report higher stress.

**Conclusions:**

BRFs cluster together, however, such clustering is not a clear-cut, all-or-none phenomenon. Students with BRFs consistently exhibited higher levels of depressive symptoms and stress. Educational and motivational interventions should target at-risk individuals including those with insufficient income or living with roommates or alone.

**Supplementary Information:**

The online version contains supplementary material available at 10.1186/s12889-024-18421-0.

## Background

Transition to university corresponds with a crucial time of psychosocial stressors including separation from family home, pressures associated with academic work, and unhealthy lifestyle habits [1]. Mental well-being can be affected in early adulthood during the university years, rendering students’ mental well-being a potential concern [2–4]. For instance, a study in the USA found that pre-veterinary students’ mental well-being declined as they progressed in their undergraduate careers [5].

The mental well-being of university students is extremely important, as it is crucial for their academic achievement and social progress, and for the economic development and success of the country [6]. Two common conditions that are frequently encountered by students are depressive symptoms and stress [7, 8].

In terms of depressive symptoms, a systematic review found that their prevalence among students ranged from 1.4 to 73.5% [9]. Others reported that 60.1% of the students surveyed across 32 distinct degree programs had mild to severe depressive symptoms in Italy [10], and depressive symptoms were present in 46% of female [11] and 37% male respondents [12] in North American medical schools. Presence of depressive symptoms among students is a crucial determinant of their academic and social functioning [13].

Likewise, students also experience high stress levels [14]. For nursing students, practicing in clinical settings was a major stress [15]; and in the UK, students reported stress [16]. University students’ stress levels are important as it can have negative academic, emotional and health outcomes, and students might employ different unhealthy strategies to cope with stress (e.g., alcohol, smoking, illicit drug/s use, unhealthy eating) [17–19].

In addition, an interplay between stress and depressive symptoms has been suggested. Students with a history of depression were more likely to experience high stress levels [20]; and while mild stress can be associated with a positive effect on students by posing alternative solutions to problems, and enhancing motivation, high stress levels are associated with depression [21, 22].

Similarly, the interplay between mental well-being and lifestyle habits is important. For instance, starting university negatively influenced students’ well-being, physical activity (PA) levels, and diet quality [23]; and a survey of freshmen students across five European countries found that stress and depressive symptoms were associated with problem drinking [24]. Likewise, among students with high levels of depressive symptoms, moderate or vigorous PA was associated with less depressive symptoms [25].

Sociodemographic characteristics also play a role. The incidence of common mental health problems differs significantly by sociodemographic characteristics such as sex, age, and living place during university time [26]. For instance, the prevalence and levels of depressive symptoms among female students were significantly higher than among men [27]; and stress levels were higher among female students than males [28]. As for accommodation during the semester, living outside the parental home in student dormitories, on campus, or in private homes, whether with roommates or alone, brings less exposure to parental control and more frequent exposure to peer influence, and thus opportunities to engage in unhealthy behaviors such as drinking alcohol, or tobacco and other drug/s use [29–31].

However, the literature reveals knowledge gaps. To the best of our knowledge, we are not aware of studies of university students in Finland that assessed the relationships between harmful lifestyle behavioral risk factors (BRFs) e.g., smoking, problematic alcohol consumption, low PA, and unhealthy nutrition patterns on the one hand; and depressive symptoms and stress on the other, employing such lifestyle BRFs and using cluster analysis (CA) to categorize students into clusters, before appraising the associations of such clusters with depressive symptoms and stress.

CA is used to identify subgroups of cases based on shared characteristics in heterogeneous samples and combines them into homogeneous groups. It provides a great deal of information about the types of cases and the distributions of variables [32]. CA is viewed as a quantitative complement to traditional linear statistics that emphasizes diversity and ecological context of behavior rather than central tendencies and simple interactions and is more person-centered and of stronger methodological rationale; nevertheless, traditional approaches are more frequently used in BRF research [33, 34]. Given that lifestyle BRFs do not occur in isolation from each other, CA is a sound method that is increasingly being employed to group together university students with similar lifestyle behaviors [35, 36].

The current study bridges these knowledge gaps. The aim of the study was to appraise the relationships between clusters of lifestyle BRFs and depressive symptoms and stress. The specific objectives were to: (1) assess four lifestyle BRFs (tobacco, smoking, problematic alcohol use, dietary habits, PA), and group students correspondingly into clusters; (2) compare the socio-demographic features of students in the generated clusters; and, (3) appraise the relationships between the generated BRFs clusters and depressive symptoms and stress.

In this paper, we use the WHO definitions of depressive symptoms (involves a depressed mood or loss of pleasure or interest in activities for long periods of time) and of stress (a state of worry or mental tension caused by a difficult situation) [37, 38].

## Methods

### Ethics, Sample, procedures

The study was approved by the Research and Ethics Committee at the University of Turku, Finland (Approval # Lausunto 10/2010) with an informed consent waiver. An email invitation with the research objectives was sent to all first, second- and third-year undergraduate students (n = 4387) enrolled at all faculties of the university, inviting them to participate. A university-wide English-language online survey was used to collect data during the academic year 2013–2014. As skills in English are generally good among young adults in Finland and particularly among university students, translation of the questionnaire into Finnish was not considered necessary. Students from all the seven faculties of the University of Turku (Humanities, Mathematics and Natural Sciences, Medicine, Law, Social Sciences, Education, and Economics) were invited. Participation was voluntary and anonymous, and data were confidential and protected (anonymous, no identifiers, strict access only to the research team, secure computer storage, password updated and regularly changed every month, no paper copies). Students were provided with information about the study as well as contact details for any questions and were informed that by completing the questionnaire, they were providing consent to participate in the study. Both the initial invitation to participate and the subsequent reminder emails fourteen days later were sent to all undergraduate students. In addition, three posters about the study were displayed in the university’s student café and the reminder was announced on the University intraweb site. Webropol platform was used for the online survey and all students received a link to the questionnaire. After completing the survey, students' answers were automatically saved and forwarded to the university’s Student Management Office. The Student Management Office gathered the completed online responses, and the data was entered electronically into an Excel spreadsheet to ensure a high level of quality assurance. Once this stage was completed, the data was forwarded to the research team.

### Research tool: Survey Questionnaire

Socio-demographic information included students’ sex and age. Subjective financial situation was measured by a single item: “How sufficient is your income?”, with a 4-point response scale, subsequently dichotomized into (always /mostly sufficient vs. always/mostly insufficient) [24]. Students were also asked about the type of accommodation during the semester, and responses were dichotomized into “I live with my parents/ partner” vs “with roomates/alone” [29]. The rationale is that living with a partner/ parents may involve compromise and need to respect one another’s boundaries and preferences which may lead individuals to engage in fewer risky behaviors; while living in student dormitories, in private flats, either with roommates or alone, entails less exposure to parental control and more exposure to peer influence and therefore to opportunities to engage in problematic behavior [39, 40].

***Perceived stress*** was measured with the four-item version of Cohen’s perceived stress scale (PSS) that measures the degree to which situations in one’s life over the past month are appraised as stressful [41]. The questions are of a general nature and items are designed to detect how unpredictable, uncontrollable, and overloaded respondents find their lives, e.g. “How often have you felt that you were unable to control the important things in your life?”; and, “How often have you felt confident about your ability to handle your personal problems?”. Students responded on a five-point scale (0 = “never”, 1 = “almost never”, 2 = “sometimes”, 3 = “fairly often”, 4 = “very often”). The PSS score was obtained by summing up answers to individual questions. Items were recoded so that higher scores indicated more perceived stress. Cronbach’s alpha coefficient was 0.75.

***Depressive symptoms*** were measured using a modified version of the Beck Depression Inventory (M-BDI) [42]. Participants were asked to describe how often they experienced 20 depressive feelings during the past few days with 6-point scale responses (from 0=”never” to 5 = “almost always”). Sample items include: “I feel sad”, “I feel I am being punished”, “I have thoughts of killing myself”, and “I have lost interest in other people”. The M-BDI score is obtained by summing up answers to individual questions. The scale showed good level of reliability. Cronbach’s alpha coefficient was 0.94.

***Problem drinking*** was assessed using CAGE screening test for problem alcohol use, consisting of four questions (Have you ever felt you should Cut down on your drinking? Have people Annoyed you by criticizing your drinking? Have you ever-felt bad or Guilty about your drinking? Have you ever had a drink in the morning to get rid of a hangover? (Eye opener). Each item has 2 response options (“Yes,” “No”) [43]. Two or three affirmative answers suggest problem drinking. We classified the respondents as non-problematic drinkers (less than two positive answers) and problematic drinkers (two or more positive answers).

***Smoking*** was measured with the item “Within the last 3 months, how often did you smoke (cigarettes, pipes, cigarillos, cigars)?” with response options “Daily,” “Occasionally,” and “Never” [44].

***Dietary assessment*** (12 items): respondents self-reported their dietary habits in a food frequency questionnaire, which included 12 variables assessing their consumption of sweets, cakes/crackers, fast food and canned foods, fresh fruit, raw and cooked vegetables and salads, meat and fish, dairy products and cereals. In the initial question “How often do you eat the following foods?“, students were asked about the frequency of their usual consumption of each food group separately (5-point scale: “several times a day”, “daily”, “several times a week”, “1–4 times a month”, “never”). The question elicited information on student’s overall food consumption. The instrument was based on pre-existing food frequency questionnaires, adapted and used in previous studies [45].

***Dietary guideline adherence score*** was calculated based on students’ responses to the food frequency questionnaire [45]. There are no specific guidelines for sweets, cakes/cookies, snacks, fast food/canned foods and sodas/soft drinks, so “1–4 times per month” and “never” were used as the recommended values. We used the above composite food intake pattern score (sweets, cakes/cookies, and snacks score) to assess sweets, cakes/cookies, and snacks combined, and healthy eating was considered present if this score was ≤ 6, corresponding to intake of these items “less often than 1–4 times a month” for each food item. Each of the fast food/canned food and lemonade/soft drink items was included as a separate item in the calculation of the objective dietary guideline adherence score. For other food groups, the WHO recommendations for the European region were used [46]. Subsequently, for the number of daily servings of fruit, raw and cooked vegetables, the cut-off value was “daily” or “several times a day”; and for meat, the cut-off was “less than daily” and for fish, it was “several times a week”. Milk and cereals were not included in the calculation of the dietary guideline adherence score because information on milk and cereals was generally too non-specific to be categorized as healthy/unhealthy. The dietary guideline adherence score (Supplementary File 1) has a maximum of 8 points calculated from the recommendations of 8 food groups: (1) sweets, cookies, snacks; (2) fast food/canned food; (3) lemonade/soft drinks; (4) fruit; (5) salad, raw vegetables; (6) cooked vegetables; (7) meat; and (8) fish [44, 46].

***Two forms of physical activity*** (i.e., vigorous PA, moderate PA) were assessed using the questions, “On how many of the past 7 days did you: (1) participate in vigorous exercise of ≥ 20 min; (2) participate in moderate exercise of ≥ 30 min?” For each form of PA, students reported the number of days they engaged in such activity (range 0–7 days). Moderate-to-vigorous PA (MVPA) was calculated by combining moderate-intensity PA and vigorous-intensity PA [47].

### Statistical analysis

Quantitative variables were presented as mean ± standard deviation, while qualitative variables were presented as frequency and percent. Independent samples t-test compared quantitative variables, while Pearson chi-square test compared qualitative variables. Two-step cluster analysis was applied to 4 BRFs (tobacco smoking, alcohol drinking, PA, eating behavior) to identify clusters that differed in criterion variables within the dataset, and the procedure combined pre-clustering and hierarchical methods. A log-likelihood distance measure was used in the two-step cluster analysis because the BRFs comprised continuous and categorical variables. Cluster number selection was automated using the Schwarz Bayesian criterion. Within each cluster, the frequency of categories and percentages were reported for categorical BRFs, whereas mean ± standard deviation were reported for continuous BRFs. Differences in the distribution of sociodemographic characteristics and BRFs across clusters were tested using Chi-square tests for categorical variables or independent samples t-tests for continuous variables. Two separate multiple linear regression models examined the association between cluster membership and depressive symptoms and stress while adjusting for participant’s sex, income sufficiency, and accommodation during semesters. Any missing values were not imputed. The number of missing values was negligible, hence we decided to use complete case analysis which limits the analysis to respondents with complete data [48]. Statistical analyses were performed using SPSS v25.0 and statistical significance was set at *p* < 0.05.

## Results

### Characteristics of the sample

The total number of responses was 1179 (response rate = 27%). Mean age of the students was ≈ 23 (SD 5) years and 823 (70.4%) were female. More than half the respondents reported always/mostly sufficient income (Table 1). During university semesters, about a third of the students lived with parents or partners. Daily smoking was rare (about 6%), and mean MVPA was 4.27 ± 3.27 days/week. Sex differences were apparent as significantly more females exhibited non-problematic drinking and had healthier eating habits.

**Table 1:** General sociodemographic and behavioral characteristics of the participants

Variable	All participants *N* = 1179	Male *n* = 346	Female *n* = 823	p-value
**Sociodemographic characteristics**				
Age (years) *	22.96 ± 5.21	22.83 ± 4.36	23.01 ± 5.55	0.59
Perceived income sufficiency				0.30
Always/ Mostly sufficient	675 (56.8)	207 (59.8)	466 (56.6)	
Always/ Mostly insufficient	487 (41)	135 (39)	348 (42.3)	
Accommodation during semester				0.40
With parents /partner	394 (33.1)	109 (31.5)	280 (34)	
Alone/ with roommates	795(66.9)	237(68.5)	543(66.0)	
**Behavioral risk factors**				
Smoking (past 3 months) (*n* = 1168)				0.23
Never	911 (76.6)	257 (74.9)	648 (79.2)	
Occasionally	183 (15.7)	63 (18.4)	119 (14.5)	
Daily	74 (6.3)	23 (6.7)	51 (6.2)	
Problem drinking (CAGE score) (*n* = 1138)				***0.014***
Non-problem drinking	810 (71.2)	218 (66.1)	588 (73.3)	
Problem drinking	328 (28.8)	112 (33.9)	214 (26.7)	
Physical activity* (days/ week) (*n* = 1157)				
MVPA	4.27 ± 3.27	4.31 ± 3.49	4.24 ± 3.18	0.75
Nutrition habits* (*n* = 1160)				
Dietary guideline adherence index	4.84 ± 1.57	4.22 ± 1.54	5.10 ± 1.51	***< 0.001***

Cell values represent frequency (%) except where indicated, * Cell values represent mean ± SD, *MVPA* Moderate to vigorous physical activity, Bold italicized cells indicate statistical significance, numbers might not sum up to total because of missing values

### Clustering of four behavioral risk factors among students

The silhouette value of cohesion and separation was ≈ 0.7 indicating good cluster quality. A total of 82 participants with missing values in the items used for the cluster analysis were not included, reducing the sample size to 1097. Cluster analysis of the four BRFs resulted in three well-defined clusters, namely Cluster 1 (Healthy Group, *n* = 649), Cluster 2 (Smokers, *n* = 245), and Cluster 3 (Nonsmokers but Problem Drinkers, *n* = 203). There were significant differences across the clusters for some of the sociodemographic characteristics (Table 2). Age and sex were not different across the clusters, however, Cluster 1 and Cluster 3 comprised significantly more respondents who reported always/ mostly sufficient income and lived with their parents/ partner during semester time.

The clusters exhibited significant differences across most of the BRFs. However, all students in Clusters 1 and 3 had never smoked, but all Cluster 2 students were occasional/daily smokers. Although problematic drinking increased from Cluster 1 (0%) to Cluster 3 (100%), Cluster 3 respondents had significantly healthier nutrition habits than both other clusters. MVPA was not significantly different between the 3 clusters, however an additional post-hoc test revealed a significant difference in MVPA between Cluster 1 and Cluster 2 (*p* = 0.02).

**Table 2:** Comparison of sociodemographic characteristics and self-rated behavioral risk factors of three clusters of university students in Finland (*N* = 1097)

Characteristic	Cluster 1 *n* = 649	Cluster 2 *n* = 245	Cluster 3 *n* = 203	p-value
**Sociodemographic**				
Age (years)	22.95 ± 5.67	22.86 ± 4.01	23.03 ± 4.45	0.94
Sex				
Female	470 (72.4)	165 (67.3)	139 (68.5)	0.23
Male	175 (27.0)	79 (32.2)	63 (31.0)	
Perceived income sufficiency				***0.009***
Always/ Mostly sufficient	387 (59.6)	118 (48.2)	118 (58.1)	
Always/ Mostly insufficient	254 (39.1)	123 (50.2)	84 (41.4)	
Accommodation during semester				***0.009***
With parents/ partner	237 (36.5)	63 (25.7)	70 (34.5)	
other	412 (63.5)	182 (74.3)	133 (65.5)	
**Behavioral risk factors**				
Smoking (past 3 months)				***< 0.001***
Never	649 (100)	0 (0)	203 (100)	
Occasionally	0 (0)	173 (70.6)	0 (0)	
Daily	0 (0)	72 (29.4)	0 (0)	
Problem drinking (CAGE score)				***< 0.001***
Non-problem drinking	649 (100)	132 (53.9)	0 (0)	
Problem drinking	0 (0)	113 (46.1)	203 (100)	
Physical activity* (days/week)				
MVPA	4.41 ± 3.39	3.84 ± 3.05	4.13 ± 3.05	0.059
Healthy eating* (points)				
Dietary guideline adherence score	4.78 ± 1.54	4.78 ± 1.64	5.17 ± 1.49	***0.005***

Cell values represent frequency (%), except where indicated, * Cell values represent mean ± SD, *MVPA* Moderate to vigorous physical activity, Bold italicized cells indicate statistical significance, numbers might not sum up to total because of missing values

### Associations of behavioral risk factor clusters with depressive symptoms and perceived stress

For the regression analysis, Table 3 depicts that two sociodemographic characteristics (female sex, income insufficiency) were significantly associated with both depressive symptoms and perceived stress. Males and those with sufficient income were significantly less likely to report depressive symptoms or stress. Accommodation status during the semester was significantly associated with depressive symptoms only, those living with parents/partner were significantly less likely to experience depressive symptoms. Compared to Cluster 1, students in the two other clusters were significantly more likely to report higher depressive symptoms. However, only Cluster 2 was significantly more likely to be associated with higher perceived stress.

**Table 3:** Associations of behavioral risk factor clusters with depressive symptoms and perceived stress

Variable	Depressive symptoms		Perceived stress	
	Std-β	β (95% CI)	Std-β	β (95% CI)
**Controlling variables**				
Sex (male)	-0.15	-6.22 (-8.52; -3.91) ^*a*^	-0.08	-0.54 (-0.94; -0.15) ^*b*^
Income sufficiency (sufficient)	-0.12	-4.60 (-6.73; -2.47) ^*a*^	-0.12	-0.77 (-1.13; -0.40) ^*a*^
Accommodation (with parents/partner)	-0.06	-2.44 (-4.66; -0.21) ^*c*^	-0.04	-0.29 (-0.68; 0.09)
**Predictors**				
Cluster 2 (vs. Cluster 1)	0.1	4.57 (1.89; 7.26) ^*a*^	0.09	0.69 (0.23; 1.15) ^*b*^
Cluster 3 (vs. Cluster 1)	0.07	3.24 (0.39; 6.09) ^*c*^	0.04	0.33 (-0.16; 0.82)

*Std-ß* standardized beta coefficient, *ß* beta coefficient, *CI* confidence interval, models adjusted for gender, income perception, accommodation during semester, ^*a*^*p* < 0.001, ^*b*^*p* < 0.01, ^*c*^*p* < 0.05

## Discussion

University students are at a critical stage of their lives, transitioning into adulthood with its unique challenges that can impact their lifestyle and health behaviors. BRFs among students refer to behaviors that can increase their risk of developing negative health outcomes [49]. Hence, there have been calls to actively provide vulnerable students with the support required to manage their mental well-being [50]. To the best of our knowledge, this study is the first to cluster four BRFs among a large sample of university students in Finland, and to weigh up the links of the clusters with depressive symptoms and stress.

Our main findings revealed three distinct BRFs clusters, with significant differences across almost all the BRFs under examination. Cluster 1 (Healthy Group) comprised students with healthier lifestyle habits who did not smoke and had no problematic drinking. On the other hand, Cluster 2 (Smokers) included occasionally/ daily smokers and almost half were problem drinkers; and Cluster 3 (Nonsmokers but Problem Drinkers) comprised 100% problematic drinkers.

These findings confirm that lifestyle BRFs do not appear in a solitary manner and do not transpire in isolation from each other. Rather, they cluster together in constellations, where individuals engaging in one risky behavior are more likely to engage in other risky behaviors. Conversely, students with healthier lifestyles are likely to maintain healthy diets, not smoke and have no problematic drinking. Except for Cluster 1, the other two clusters represented students with 50% and 100% problematic alcohol consumption. It could be that for these young adults at this stage of life within a university setting characterized by a heightened sense of fraternity, excessive drinking patterns might be part of the student life [51].

The current study assessed the relationships between the BRFs clusters and depressive symptoms and stress after adjusting for sex, income sufficiency and accommodation during semesters. In terms of gender, the current findings demonstrate that males were significantly less likely to report depressive symptoms and stress, congruent with a body of evidence among students in several countries [28, 52]. The so-called gender paradox in health is that women live longer than men but have more chronic and mental health problems throughout the life course [53]. A recent study assessed sex differences in mental well-being using items that included feeling unhappy or depressed, having lost confidence in oneself and being unable to overcome one’s problems [54]. This study found that sex differences in mental well-being in the Nordic countries are not particularly small and also remain when other social and lifestyle factors are considered [54]. Similarly, another recent study on loneliness, mental well-being, and self-esteem among adolescents in four Nordic countries (Denmark, Finland, Iceland, Sweden) found the prevalence of positive mental well-being among boys was higher than girls; boys had higher self-esteem compared to girls; and feelings of loneliness were more frequent among girls [55]. Such existence of poorer health outcomes and gender differences in mental well-being within Nordic countries despite their robust welfare systems and gender equality policy, has been proposed to be a reflection of complex societal influences [54].

As regards income sufficiency and accommodation during the semester, students with sufficient income were less likely to report depressive symptoms and stress, congruent with studies where perceived socioeconomic status (SES) predicted mental and general well-being [56, 57]. Although SES is not the sole predictor of mental well-being, its impact can help to identify at-risk populations and inform policy decisions aimed at reducing health disparities. In addition, living with parents/partners was protective for depressive symptoms in the current study. This is supported by other evidence where young adults living with parents/partners reported fewer depressive symptoms compared to those who live alone/with roommates, as living with parents/partner can provide a sense of security, financial stability, social and emotional support, and a sense of belonging which positively impacts mental well-being [58].

### Associations between BRF clusters and stress

Compared to Cluster 1 (Healthy Group), Cluster 2 (Smokers) who also exhibited some problem drinking and had significantly lower MVPA, were more likely to report higher stress (*p* < 0.01), even after adjusting for the three potential confounders. This is congruent with a raft of studies where the use of alcohol or other substances, as well as the presence of more substance-related problems, were associated with higher stress [24, 59]. Tension reduction theory holds that tension-producing circumstances (i.e., stressors) might lead to increased drinking, as alcohol is perceived to reduce tension and therefore increased tension (strains or stress) may cause drinking [60]. In addition, regular PA, whether moderate or intense, helped to reduce stress [61], improve mood [62] and sleep quality [63], all important for managing stress.

Such differences that we identified between Cluster 1 and Cluster 2 in terms of the association of the latter with higher stress were not observed when comparing Cluster 1 (Healthy Group) with Cluster 3 (Nonsmokers but Problem Drinkers). Healthy eating habits among the risk-taking Cluster 3 students may serve as protective factor against perceived stress, supporting studies that found stress was associated with unhealthy eating behavior changes [64, 65].

### Associations between BRF clusters and depressive symptoms

Compared to Cluster 1 (Healthy Group), the two other less healthy clusters were significantly more likely to be associated with higher depressive symptoms after adjusting for sex, income sufficiency and accommodation during semesters. These findings are consistent with research of college students that found relationships between depressive symptoms and various BRFs such as problematic drinking [24] or sedentary behavior and physical inactivity [66].

The association between cluster membership and depressive symptoms exhibited a *p* value of < 0.05 when Cluster 1 was compared with Cluster 3 (Nonsmokers but Problem Drinkers, but simultaneously also physically active). However, when Cluster 1 (Healthier group) was compared with Cluster 2 (Smokers who also simultaneously exhibited the least PA), the significance level increased (*p* < 0.001). This suggests that the relationship between cluster and depressive symptoms was more pronounced among Cluster 2 students. As highlighted above, PA might have numerous mental well-being benefits, including reducing the risk of developing depressive symptoms, as regular exercise helps to improve mood, reduce stress, and increase the release of the natural mood-enhancing endorphins in the brain [67–69]. Therefore, a sedentary lifestyle and lack of PA can increase the risk of depressive symptoms.

This study has limitations. The survey was cross-sectional, so the direction of the association between BRFs and depressive symptoms and stress cannot be ascertained. A point to note is that BRFs such as physical inactivity or problematic drinking can be a consequence of stress or depressive symptoms [70], although the relationship has been suggested to be bi-directional, where sedentary behavior and drinking might also lead to depressive symptoms [71]. Data were self-reported, with possible recall, social desirability/ sociability biases; and the response rate was not very high which is quite common with internet-based surveys [72] and could negatively impact representativeness of a sample which in turn affects the internal validity and limits generalizability of findings [73]. As we were unable to obtain data about those who did not participate in the survey, we could not assess differences between students who participated in the survey and those who did not.

The study has many strengths, including a large sample of students from across all the university departments/faculties categorized into clusters, reporting on a wide range of BRFs pertinent to health, thus extending previous studies that focused on a single/few health behavior(s). It is the first study among university students in Finland that appraised and categorized students into BRFs clusters and explored the associations of the clusters with two mental well-being indicators, whilst controlling for several potential confounders. In the questionnaire, an item regarding problems related to completion of the survey instrument in English was included, but according to the responses, almost none of the respondents reported serious difficulties understanding any of the questions.

## Conclusion

BRFs include problematic drinking, smoking, low PA, and unhealthy dietary patterns. These risk factors usually do not occur in isolation but rather tend to cluster together, creating congregations that are associated with depressive symptoms and stress among university students. Fortunately, BRFs are a product of lifestyle choices, and therefore can be potentially modified through effective behavioral modification interventions. Our findings are important to educators, policymakers and other stakeholders involved with these young adult populations. Prevention and intervention efforts could focus on risk groups (e.g., students with insufficient income, living with roommates or alone) and on implementing effective educational and motivational interventions to encourage regular PA, healthy eating habits and nutrition, as well as smoking cessation and responsible drinking programs.

### Electronic supplementary material

Below is the link to the electronic supplementary material.

Supplementary Material 1

## Data Availability

Data are available from the authors upon reasonable request to corresponding authors.
