# Supplementary material for: Clusters of lifestyle behavioral risk factors and their associations with depressive symptoms and stress: evidence from students at a university in Finland
Source: BMC Public Health. 2024 Apr 22;24:1103. doi: 10.1186/s12889-024-18421-0 (PMC11034152; doi:10.1186/s12889-024-18421-0)
Supplement: Supplementary file 1 — Supplementary Material 1 [file 12889_2024_18421_MOESM1_ESM.docx]

**Supplementary File 1**

**Computation of Dietary Guideline Adherence Score**

***Dietary guideline adherence score*** with maximum of eight points (eight guidelines) was computed, from eight foods based on responses from the food frequency questionnaire.: (1) sweets, cookies and snacks; (2) fast food/ canned food; (3) lemonade/soft drinks; (4) fruits; (5) salad and raw vegetables; (6) cooked vegetables; (7) meat; and (8) fish

Calculation for sweets, cakes and snacks was based on sum score of these items from FFQ.

When the sum score of these items was ≤ 6, we consider it as the recommended (recoded as 1 in SPSS). It corresponds to intake of these items “less often than 1–4 times a month”.

For calculation was for fast food/canned food, we employed “1–4 times a month” and “never” responses from FFQ as recommended (recoded as 1 in SPSS).

For calculation of lemonade/soft drinks, we employed “1–4 times a month” and “never” as recommended (recoded as 1 in SPSS).

For calculation of fruit, raw and cooked vegetables, we used "daily" or "several times a day" as recommended (recoded as 1 in SPSS).

For calculation of meat, the cut-off was "less than daily" (recoded as 1 in SPSS).

For calculation of fish, we used "several times a week" as recommended (recoded as 1 in SPSS).
